# Optimal time for COVID-19 vaccination in rituximab-treated dermatologic patients

**DOI:** 10.3389/fimmu.2023.1138765

**Published:** 2023-03-15

**Authors:** Chutima Seree-aphinan, Yanisa Ratanapokasatit, Poonkiat Suchonwanit, Ploysyne Rattanakaemakorn, Pichaya O-Charoen, Prapaporn Pisitkun, Thanitta Suangtamai, Chavachol Setthaudom, Sonphet Chirasuthat, Kumutnart Chanprapaph

**Affiliations:** ^1^ Division of Dermatology, Department of Medicine, Faculty of Medicine, Ramathibodi Hospital, Mahidol University, Bangkok, Thailand; ^2^ Division of Allergy, Immunology, and Rheumatology, Department of Medicine, Faculty of Medicine, Ramathibodi Hospital, Bangkok, Thailand; ^3^ Immunology Laboratory, Department of Pathology, Faculty of Medicine, Ramathibodi Hospital, Mahidol University, Bangkok, Thailand

**Keywords:** rituximab, anti-CD20 antibody, vaccines, COVID-19 vaccines, immunogenicity, immune-mediated dermatologic diseases, autoimmune diseases

## Abstract

**Background:**

By depleting circulating B lymphocytes, rituximab time-dependently suppresses coronavirus disease 2019 (COVID-19) vaccines’ humoral immunogenicity for a prolonged period. The optimal time to vaccinate rituximab-exposed immune-mediated dermatologic disease (IMDD) patients is currently unclear.

**Objective:**

To estimate the vaccination timeframe that equalized the occurrence of humoral immunogenicity outcomes between rituximab-exposed and rituximab-naïve IMDD patients.

**Methods:**

This retrospective cohort study recruited rituximab-exposed and age-matched rituximab-naïve subjects tested for severe acute respiratory syndrome coronavirus 2 (SARS-CoV-2)-specific immunity post-vaccination. Baseline clinical and immunological data (i.e., immunoglobulin levels, lymphocyte immunophenotyping) and SARS-CoV-2-specific immunity levels were extracted. The outcomes compared were the percentages of subjects who produced neutralizing antibodies (seroconversion rates, SR) and SARS-CoV-2-specific IgG levels among seroconverters. The outcomes were first analyzed using multiple regressions adjusted for the effects of corticosteroid use, steroid-spearing agents, and pre-vaccination immunological status (i.e., IgM levels, the percentages of the total, naïve, and memory B lymphocytes) to identify rituximab-related immunogenicity outcomes. The rituximab-related outcome differences with a 95% confidence interval (CI) between groups were calculated, starting by including every subject and then narrowing down to those with longer rituximab-to-vaccination intervals (≥3, ≥6, ≥9, ≥12 months). The desirable cut-off performances were <25% outcome inferiority observed among rituximab-exposed subgroups compared to rituximab-naïve subjects, and the positive likelihood ratio (LR+) for the corresponding outcomes ≥2.

**Findings:**

Forty-five rituximab-exposed and 90 rituximab-naive subjects were included. The regression analysis demonstrated a negative association between rituximab exposure status and SR but not with SARS-CoV-2-specific IgG levels. Nine-month rituximab-to-vaccination cut-off fulfilled our prespecified diagnostic performance (SR difference between rituximab-exposed and rituximab-naïve group [95%CI]: -2.6 [-23.3, 18.1], LR+: 2.6) and coincided with the repopulation of naïve B lymphocytes in these patients.

**Conclusions:**

Nine months of rituximab-to-vaccination interval maximize the immunological benefits of COVID-19 vaccines while avoiding unnecessary delay in vaccination and rituximab treatment for IMDD patients.

## 1 Introduction

During the pre-vaccination period, the coronavirus disease 2019 (COVID-19) pandemic substantially burdened the world medically and socioeconomically, with the major determinant of patients’ survival relying solely on supportive measures (1). By inducing certain inflammatory cascades (2), COVID-19 vaccination induces the production of pathogen-specific neutralizing antibodies (3), which plays a significant role in decelerating the disease spreading and reducing the cases of COVID-19-related organ failure, hospitalization and mortality, allowing a gradual resume of pre-pandemic lifestyle in many parts of the world (4, 5). Nonetheless, emerging COVID-19 variants and the spike in disease incidence associated with waning immunity emphasize the importance of maintaining high-level immunity to prevent the COVID-19 pandemic from rehappening (6, 7). Achieving this goal can be challenging for patients who require rituximab treatment, as numerous studies demonstrated markedly decreased humoral immunogenicity of COVID-19 vaccines in these patients, possibly resulting in an increased susceptibility to infection than the general population (8–10). In addition, given their comorbidities and immunosuppressed status, managing severe COVID-19 in these patients can be extremely challenging both in the diagnosis (11) and treatment aspects (1, 12), making it necessary to maximize protection *via* adequate passive immunity (13–15). With the increasing indications of rituximab treatment in dermatological conditions (16–19), immune-mediated dermatologic disease (IMDD) patients are among those whose immune responses to COVID-19 vaccines can be affected.

Our previous study showed that rituximab use was associated with non-seroconversion following COVID-19 vaccination among autoimmune bullous disease patients (20). Avoiding vaccination while rituximab is taking effect, whereby completing vaccination 2-4 weeks before rituximab treatment, is suggested by international practice guidelines (21, 22). However, this recommendation may not be practicable for some IMDD patients, such as patients with severe pemphigus, as their disease circumstance may benefit from urgent rituximab treatment (23, 24). For IMDD patients who need to be vaccinated after drug administration, the optimal time for COVID-19 vaccination is still being determined. Therefore, this study aimed to determine the earliest rituximab-to-vaccination interval cut-off for successful vaccination among rituximab-treated IMDD patients by comparing the COVID-19 vaccine’s humoral immunogenicity outcomes measured from rituximab-exposed subjects with various rituximab-to-vaccination intervals to those of rituximab-naïve individuals.

## 2 Materials and methods

### 2.1 Study design and patient selection

We employed a cohort study design to estimate the vaccination timeframe that equalized the immunogenicity outcomes between rituximab-exposed and rituximab-naïve IMDD patients. This retrospective pilot cohort study screened participants of previous prospective cohort studies conducted in our institutions for eligibility. These studies evaluated either or both humoral and cellular immune responses to various types of COVID-19 vaccines in IMDD patients 28 days post-vaccination (20, 25). Adult (≥ 18 years old) patients diagnosed with IMDD or cutaneous manifestations of connective tissue diseases (e.g., systemic lupus erythematosus with cutaneous lupus erythematosus, rheumatoid arthritis with cutaneous vasculitis, dermatomyositis, systemic sclerosis, mixed connective tissue disease) tested for SARS-CoV-2-specific humoral immunity levels after vaccination were included in this study. Subjects receiving at least one course of rituximab per any standard protocols before vaccination were included as an exposed group (26). Subjects who had never received rituximab were recruited as the unexposed group. A ratio between the number of rituximab-exposed to rituximab-naive subjects was set at 1:2. The two groups were matched by age groups (18-<40, 40-<60, ≥60 years old). The subjects were excluded if they received rituximab between the first dose of the vaccine and the date of the vaccine’s immunogenicity assessment. This study was in full compliance with the declaration of Helsinki and was approved by the Human Research Ethics Committee, Faculty of Medicine Ramathibodi Hospital, Mahidol University (MURA 2022/686, Thai Clinical Trails Registry No. 20221213001), which granted a waiver of consent.

### 2.2 Data sources and extraction

Data were extracted from the hospital’s electronic medical record and case record forms of the original cohort studies and transferred to new case record forms in a deidentified manner. Clinical information collected includes age, sex, the diagnosis of immune-mediated dermatologic diseases, other comorbidities, immunosuppressive drugs used listed in medical records of every clinic visit, baseline immunological assessment such as immunoglobulin levels, peripheral blood immunophenotyping before vaccination (if available). Treatment protocols and the date of the last dose received were documented for rituximab-exposed patients. COVID-19 vaccines-related data extracted comprises the types of COVID-19 vaccines received, the date of vaccination, the magnitude of humoral immune responses to COVID-19 vaccines in the forms of SARS-CoV-2-specific binding IgG or neutralizing antibody (NAb) levels, and the presence or absence of SARS-CoV-2-specific cellular immunity was demonstrated either by interferon-*γ* release assay or enzyme-linked immune absorbent spot test.

### 2.3 Operational definition of humoral immunogenicity outcomes

The primary outcome was the ability to produce the SARS-CoV-2-specific NAb (i.e., seroconversion probability). Seroconversion probability was calculated as the proportion of those who tested positive for NAb within the group and defined as seroconversion rates (SR). NAb was measured by the surrogate viral neutralization test (sVNT) (SARS-CoV-2-NeutraLISA, Euroimmun, Lübeck, Germany), which quantifies the capacity of the patient-produced NAb in inhibiting the *in-vitro* interaction between human angiotensin-converting enzyme 2 receptors and SARS-CoV-2 S1 receptor binding protein (RBD). Results were reported as the percentages of reactions inhibited ranging from 0-100% (positive threshold: 35%). The secondary outcome was the magnitude of humoral immune responses to COVID-19 vaccines in seroconverters represented by SARS-CoV-2 S1 RBD IgG level measured by chemiluminescent microparticle immunoassay (SARS-CoV-2 IgG II SEMI QUANT assay, Abbott, Chicago, US), which was more suitable than sVNT for this purpose because its limit of detection was broader than sVNT.

### 2.4 Statistical analysis

STATA 17.0 (StataCorp LLC, TX, US) was used for analysis with p < 0.05 as a statistically significant threshold. Categorical variables were summarized as percentages and continuous variables as means with standard deviations or medians with interquartile ranges (IQR) depending on data distribution. Subjects with missing outcome data were excluded prior to analysis.

We defined the rituximab-to-vaccination interval cut-offs for successful vaccination as the time when the SR of rituximab-exposed subjects was comparable to those of rituximab-naïve subjects. To identify this timeframe, we divided the analyses into two steps. First, the primary and secondary outcomes were compared between rituximab-exposed and rituximab-naïve groups using chi-squared and rank-sum tests. The immunogenicity outcomes that showed a statistically significant difference between groups subsequently underwent regression analyses adjusted for corticosteroid use, steroid-spearing agents, and other possible confounders found during univariate analysis to ensure that the differences in the occurrence of outcomes were not attributable to non-rituximab immunosuppressants. Outcomes which retained significant associations with rituximab exposure status were used for rituximab-to-vaccination interval cut-off analysis. Second, the mean or proportional differences and their 95% confidence interval (CI) in the rituximab-related immunogenicity outcomes between rituximab-exposed and rituximab-naïve groups were calculated. The desirable diagnostic performances of the cut-offs were set as follows; the immunogenicity outcomes observed among rituximab-exposed subgroups were less than 25% inferior to those of rituximab-naïve subjects, and the positive likelihood ratio for predicting the immunogenicity outcomes at least two. The comparisons were started by including every subject and then narrowed down to rituximab-exposed subgroups with longer rituximab-to-vaccination intervals, starting at ≥3 months and increasing the cut-offs sequentially at three-month intervals until reaching ≥12 months.

## 3 Results

### 3.1 Clinical characteristics of study participants

Forty-seven IMDD patients with a history of rituximab exposure before vaccination were identified from the database; 45 were included as two subjects received rituximab again before the immunogenicity assessment date. Ninety rituximab-naïve subjects were included in the unexposed group (**Table 1**). Post-vaccination SARS-CoV-2-specific S1 RBD IgG and sVNT were measured in all subjects, while SARS-CoV-2-specific cell-mediated immunity was evaluated in 61%. All rituximab-exposed subjects received rheumatoid arthritis treatment protocol (two intravenous infusions of rituximab 1,000 mg two weeks apart); the intervals between rituximab and vaccination ranged from 80 to 931 days (median [IQR]: 365 [151, 783]). Approximately 60% of the study subjects were patients with autoimmune bullous dermatoses; the rest were diagnosed with connective tissue diseases. Subjects in both groups used similar immunosuppressive therapies. The types of COVID-19 vaccines were equally distributed between groups, with the majority of subjects receiving a viral vector vaccine (i.e., ChadOx1-s recombinant). Baseline immunoglobulin level measurements and lymphocyte immunophenotyping performed in 93.3% of subjects showed more rituximab-exposed subjects with depleted B lymphocytes and hypoIgM than rituximab-naïve subjects. There was no significant difference in the overall number of peripheral blood T lymphocyte and B lymphocyte subsets between rituximab-exposed and rituximab naïve groups.

**Table 1:** Study participants.

	Rituximab-exposed (N = 45)	Rituximab-naïve (N = 90)	p
Age, median (IQR)	62.0 (51.0-70.0)	65.0 (51.0-72.0)	0.408^a^
Sex, female n (%)	27 (60.0)	68 (75.6)	0.062^b^
Immune-mediated dermatologic diseases, n (%)			0.060^b^
• Autoimmune bullous diseases	30 (66.6)	50 (55.5)	
• Systemic lupus erythematosus with cutaneous involvement	3 (6.7)	23 (25.6)	
• Rheumatoid arthritis with dermatologic manifestations	7 (15.6)	8 (8.9)	
• Other connective tissue diseases with skin involvements (e.g., dermatomyositis, systemic sclerosis, mixed connective tissue disease etc.)	5 (11.1)	9 (10.0)	
Systemic medications used, n (%)			
• Azathioprine	14 (31.1)	39 (43.3)	0.170^b^
• Mycophenolate mofetil	7 (15.6)	17 (18.9)	0.633^b^
• Prednisolone	32 (71.1)	59 (65.6)	0.516^b^
o Prednisolone dosage, median (IQR)	5 (3.5-6.3)	5 (3.3-7.5)	0.365^b^
• Methotrexate	8 (17.8)	11 (12.2)	0.382^b^
• Others immunosuppressants (e.g., leflunomide, tacrolimus)	3 (6.7)	3 (3.3)	0.376^b^
• Systemic non-immunosuppressive immunomodulators (e.g., (colchicine, hydroxychloroquine)	9 (20.0)	11 (12.2)	0.230^b^
Types of primary regimen COVID-19 vaccines, n (%)			0.211^b^
• Homologous CoronaVac	6 (13.3)	12 (13.3)	
• Homologous ChadOx1-s recombinant	32 (71.1)	73 (81.1)	
• Homologous mRNA vaccines (Pfizer or Moderna COVID-19 vaccine)	4 (8.9)	4 (4.5)	
• Heterologous inactivated-viral vector vaccines (1^st^ dose: CoronaVac, 2^nd^ dose ChadOx1-s recombinant, between-dose interval: 4 weeks)	3 (6.7)	1 (1.1)	
Baseline immunologic studies			
• B lymphocytes (% CD19+ cells among total lymphocytes), median, IQR	2.81 (0.1-9.21)	7.5 (5.4-15.9)	<0.001^a*^
o % Subjects with depleted B lymphocytes (< 1%)	11 (24.4)	2 (2.2)	<0.001^b*^
o Naïve B lymphocytes (% IgD+/CD27- cells among total CD19+ cells), median, IQR	74.6 (2.0-86.5)	54.6 (35.2-68.7)	0.335^a^
o Memory B lymphocytes (%IgD-/CD27+ cells among total CD19+ cells), median, IQR	13.9 (6.1-56.6)	22.6 (15.7-35.1)	0.314^a^
• CD4+ T lymphocytes (% CD4+ cells among CD3+lymphocytes), median, IQR	63.8 (56.0-70.9)	59.3 (50.6-69.4)	0.061^a^
• CD8+ T lymphocytes (% CD8+ cells among CD3+lymphocytes), median, IQR	29.4 (24.9-37.2)	33.4 (25.3-41.6)	0.151^a^
• % Subjects with hypoIgM (<0.4 g/L)	12 (30.8)	11 (12.6)	0.015^b*^
• % Subjects with hypoIgG (<7 g/L)	0	2 (2.3)	0.340^b^
• % Subjects with hypoIgA (<0.7 g/L)	1 (2.6)	1 (1.2)	0.557^b^
Post-vaccination SARS-CoV2-specific immunity levels			
• % Subjects tested positive for NAb	18 (40.0)	59 (65.6)	0.005^b*^
• SARS-CoV-2 S1 RBD IgG level in Nab-positive subjects, median (IQR	435.8 (155.6-769.9)	272.1 (107.2-536.8)	0.354^a^
• % Subjects tested positive for SARS-CoV-2 specific cellular immunity *via* IGRA or ELISPOT test, n (%)	21 (63.6)	25 (50.0)	0.221^b^

*p-value < 0.05  ^a^rank sum test ^b^chi square tests.

bau, international binding antibody unit; coronavirus disease 2019, COVID-19; CD, cluster of differentiation; ELISPOT, enzyme-linked immune absorbent spot test; IGRA, interferon-*γ* release assay; IQR, interquartile range; NAb, neutralizing antibody; RBD, receptor binding protein, SARS-CoV-2, severe acute respiratory syndrome coronavirus 2.

### 3.2 Immunogenicity outcomes among study participants

Overall, the primary outcome (i.e., seroconversion) was found less frequently among rituximab-exposed subjects than in the rituximab-naïve group (**Table 1**). For the secondary immunogenicity outcomes, anti-SARS-CoV-2 S1 RBD IgG levels measured from seroconverted subjects did not significantly differ between the two groups, suggesting that rituximab-exposed subjects can mount the humoral immune response to COVID-19 vaccines of similar strength to rituximab-naïve subjects once they were able to produce NAb. In 11 (24.4%) rituximab-exposed and 18 (20.0%) rituximab-naïve subjects, the attenuated humoral immune responses to COVID-19 manifested as the production of SARS-CoV-2 binding antibodies without viral neutralizing capacity (i.e., tested positive for SARS-CoV-2-specific S1 RBD IgG but negative for neutralizing antibody by sVNT). The proportion of subjects who mounted cell-mediated immunity was also similar between the two groups. Multiple logistic regression analysis demonstrated an inverse correlation between rituximab exposure status and seroconversion (correlation coefficient [95%CI]: -1.53 [-2.57, -0.51], p=0.004) after adjusted for the effects of corticosteroid dosages, the use of steroid-sparing agents, and pre-vaccination immunological status (i.e., IgM levels, the percentages of the total, naïve, and memory B lymphocytes). The analysis also showed a positive association between seroconversion and the percentage of naïve B lymphocytes (correlation coefficient [95%CI]: 0.04 [0.01, 0.08], p=0.009) but not with the percentages of total and memory B lymphocytes. Regression analyses adjusted for the same covariates did not find an association between the anti-SARS-CoV-2 S1 RBD IgG levels and rituximab exposure status. These findings concluded that SR but not anti-SARS-CoV-2 S1 RBD IgG levels as rituximab-related immunogenicity outcomes. SR was therefore used as the outcome variable during cut-off analysis.

### 3.3 Rituximab-to-vaccination intervals: cut-off analysis

**Figure 1:**
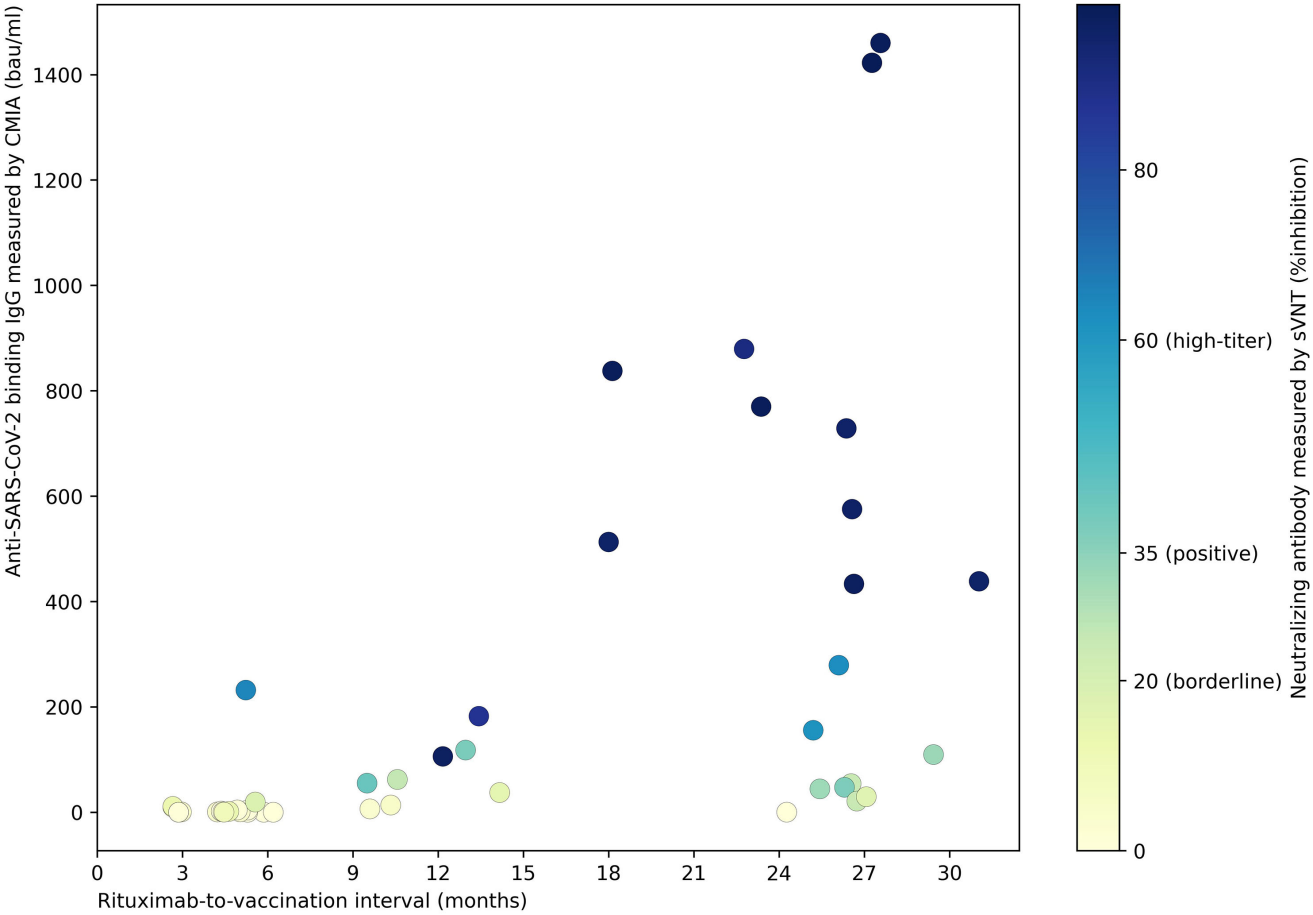
The strength of humoral immune responses^†^ to COVID-19 vaccines among rituximab-exposed IMDD patients with different rituximab-to-vaccination intervals. Post-vaccination SARS-CoV-2-specific binding and neutralizing antibody levels were demonstrated. NAb production was observed in IMDD patients vaccinated as early as six months post-rituximab administration, with a stronger humoral immune response as rituximab-to-vaccination intervals were extended. Some patients could not produce NAb despite being vaccinated two-year post-rituximab. ^†^CMIA (SARS-CoV-2 IgG II SEMI QUANT assay, Abbott, Chicago, US) measured the serum concentration of antibodies bound to SARS-CoV-2 S1 receptor binding protein. sVNT (SARS-CoV-2-NeutraLISA, Euroimmun, Lübeck, Germany) provides an *in-vitro* evaluation of the ability of anti-SARS-CoV-2 S1 IgG in the patient’s serum to prevent the interaction between SARS-CoV-2 S1 and human angiotensin-converting enzyme 2. bau, international binding antibody unit; CMIA, chemiluminescent microparticle immunoassay; IMDD, immune-mediated dermatologic diseases; NAb, neutralizing antibody; SARS-CoV-2; severe acute respiratory syndrome 2; sVNT, surrogate viral neutralization test.

**Figure 2:**
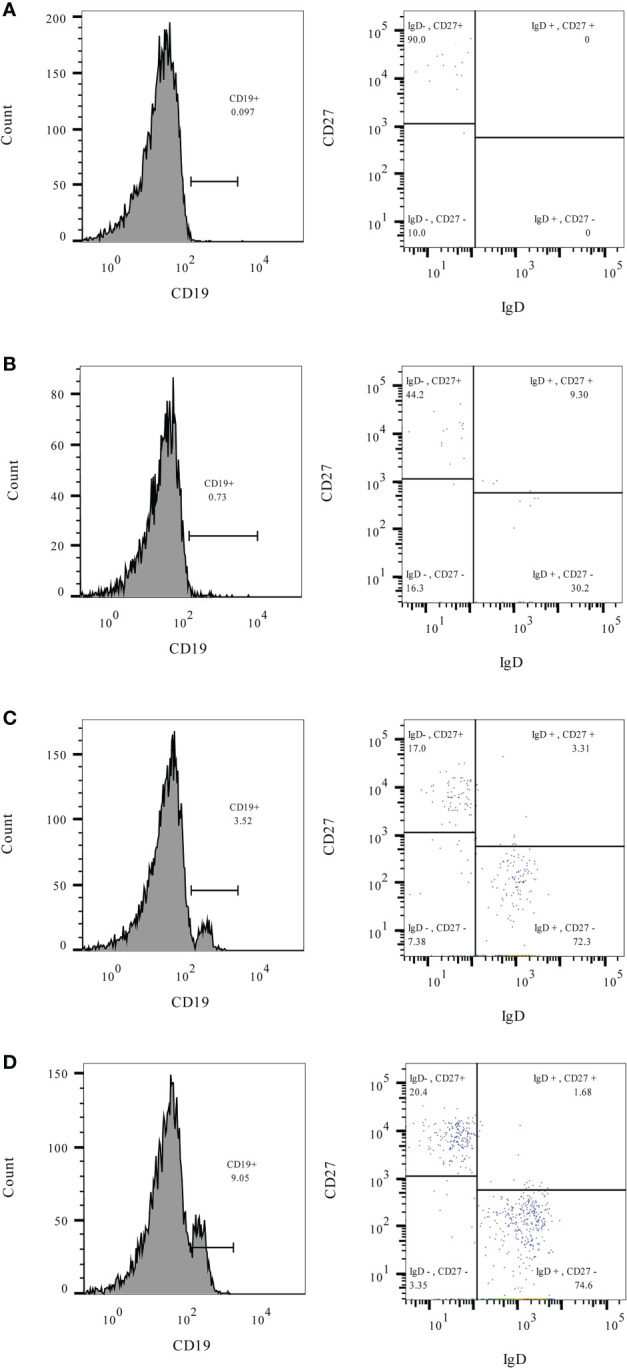
The percentages of total (CD19+), naïve (CD19+/IgD+/CD27-), and memory (CD19+/IgD-/CD27+) B lymphocytes measured before COVID-19 vaccination in rituximab-exposed subjects with different rituximab-to-vaccination intervals. Examples of pre-vaccination B lymphocyte immunophenotyping^†^ were demonstrated for rituximab-exposed subjects with rituximab-to-vaccination intervals of 3-6 months **(A)**, 6-9 months **(B)**, 9-12 months **(C)**, and more than 12 months **(D)**. Naïve B lymphocytes predominated the B lymphocyte population of subjects with rituximab-to-vaccination intervals longer than nine months. ^†^Leukocytes were prepared for flow cytometry from 25µL of fresh heparinized blood using Lyse/Stain/Wash method. Fluorescent-conjugated antibodies used for staining include C45-PE (Cat. No. 368510 Biolegend, SD, CA), CD19-PerCP-Cy5.5 (Cat. No. 45-0199-42, Thermo Fisher Scientific, WLM, MA), IgD-FITC (Cat. No. 11-9868-42, Thermo Fisher Scientific, WLM, MA), and CD27-PE-Cy7 (Cat. No. 25-0271-82, Thermo Fisher Scientific, WLM, MA). Lymphocytes were identified by size (forward scatter) and granularity (side scatter) and confirmed with surface expression of CD19 and CD45. CD19+ B lymphocytes were gated as naïve or memory subsets based on their IgD and CD27 expression patterns.

Immunogenicity outcomes among rituximab-exposed subjects were illustrated by their rituximab-to-vaccination intervals in **Figure 1**, which showed the clustering of seroconverters towards the longer rituximab-to-vaccine intervals. Most non-seroconverters completed COVID-19 vaccination within the first six-month post-rituximab administration. The first patient who was able to produce NAb was vaccinated at approximately six months after rituximab administration; the number of seroconverters gradually increased after about nine months had passed from rituximab treatment, although some patients still could not produce NAb despite being vaccinated two-year post-rituximab. Subgroup analyses determining rituximab-to-vaccination intervals cut-offs for successful vaccination demonstrated that the cut-offs of at least six months raised the likelihood ratio for seroconversion to above two (**Table 2**). Additionally, nine months or above were required for the outcome differences between rituximab-exposed and rituximab-naïve groups to decrease below 25%. The earliest rituximab-to-vaccination cut-off, which fulfilled our prespecified desirable diagnostic performance, is nine months. Examining B lymphocyte subpopulations in subjects with different rituximab-to-vaccination intervals revealed naïve cell-predominant populations among subjects with longer rituximab-to-vaccination intervals (**Figure 2**). The median [IQR] percentages of pre-vaccination naïve B lymphocytes in subjects vaccinated nine months before and after rituximab treatment were 0.8 [0-3.9] and 81.9 [68.1-87.5], respectively (p<0.001).

**Table 2:** SARS-CoV-2-specific humoral immunity levels achieved by rituximab users with different ranges of rituximab-to-vaccination intervals.

Participants subgrouped by pre-specified rituxmab-to-vaccination intervals	SARS-CoV-2 S1 RBD IgG levels^†^, bau/ml (median, IQR)	NAb level^†^ (%inhibition: 0-100%) (median, IQR)	SR (%)	SR difference [rituximab-exposed – rituximab-naïve], point estimate (95%CI)	LR+ for seroconversion
Any intervals	37.4 (1.5-231.9)	24.2 (4.5-87.0)	40.0	-25.6 (-42.9, -8.2)	0.5
≥ 3 months	50.7 (3.1-356.2)	28.8 (4.7-93.4)	45.0	-20.6 (-38.8, -2.3)	1.2
≥ 6 months	113.7 (40.9-544.1)	51.7 (24.4-96.4)	60.7	-4.8 (-25.4, 15.7)	2.3
≥ 9 months	118.0 (44.3-575.2)	61.5 (24.6-96.6)	63.0	-2.6 (-23.3, 18.1)	2.6
≥12 months	182.3 (47.3-728.5)	87.0 (32.1-97.5)	69.6	4.0 (-17.2, 25.2)	3.4

^†^SARS-CoV-2 S1 RBD IgG and NAb was measured by CMIA and sVNT respectively. CMIA (SARS-CoV-2 IgG II SEMI QUANT assay, Abbott, Chicago, US) measured the serum concentration of antibodies bound to SARS-CoV-2 S1 receptor binding protein. sVNT (SARS-CoV-2-NeutraLISA, Euroimmun, Lübeck, Germany) provides an in-vitro evaluation of the ability of anti-SARS-CoV-2 S1 IgG in the patient’s serum to prevent the interaction between SARS-CoV-2 S1 and human angiotensin-converting enzyme 2.

CI, confidence interval; IQR, interquartile range; LR+, positive likelihood ratio; NAb, neutralizing antibody; sVNT, surrogate viral neutralisation test; SARS-CoV-2, severe acute respiratory syndrome coronavirus 2; SR, seroconversion rate.

## 4 Discussion

The negative impact of rituximab on vaccines’ immunogenicity has been demonstrated for many vaccines before the arrival of COVID-19 vaccines (27–29). Despite the differences in dosage and timing of drug administration, most patients who required repeated courses of rituximab treatment, including those with IMDD, have struggled to mount humoral immunity following COVID-19 vaccines (8, 9, 20, 30–32). Attempts to rectify the situation by using more-immunogenic vaccine platforms (e.g., mRNA and viral vector technologies), lowering rituximab dose, or giving additional vaccine doses have not shown promising benefits (32–36). Previous COVID-19 vaccine immunogenicity studies involving patients with systemic autoimmune diseases, multiple sclerosis, and lymphoma suggested that the key to successful COVID-19 vaccination among rituximab users is to extend the interval between rituximab treatment and vaccination (37–39). In keeping with the previous studies, we also found a positive correlation between the longer rituximab-to-vaccination intervals and the seroconversion probability of rituximab-exposed IMDD patients.

Current evidence has placed the preferential vaccination time frame for rituximab users at ≥ 12 months after the rituximab treatment (37, 40); delaying rituximab administration to match this timeframe may not be in the patient’s best interest for IMDD patients who may require annual or biannual maintenance therapy to control the disease activity or prevent relapse (41). The critical question is, “what would be the appropriate timing to vaccinate IMDD patients after rituximab treatment?” Experts suggest waiting at least five months after the last rituximab administration (21, 22). Our study, corresponding to others, found that vaccinating at less than six months post-rituximab is extremely low yielded since the humoral immune response to COVID-19 vaccines was virtually blocked by rituximab during this period (42–45). IMDD patients who required rituximab every six months should be considered for alternative COVID-19 preventive measures, for instance, passive immunization with Tixagevimab/Cilgavimab injection (i.e., human monoclonal antibodies against the surface spike protein of SARS-CoV-2). For patients with an allowable gap of rituximab treatment longer than six months, we proposed a vaccination timeframe of at least nine months post-rituximab to balance the vaccine immunogenicity against the delay in vaccination. This cut-off was supported by two other clinical studies in rheumatic disease patients; one of them also found the reappearance of circulating naïve B lymphocytes at this time, similar to our finding (38, 46). Interestingly, based on our calculated positive likelihood ratios for predicting seroconversion, extending rituximab-to-vaccination time beyond 12 months did not substantially escalate the seroconversion rate among rituximab-exposed IMDD patients as the positive likelihood ratios remained less than five.

Although a low number of circulating CD19^+^ B lymphocytes has been proposed as a marker of vaccine non-responders (47, 48), a few studies have argued against this idea as they found that B-cell depleted patients with as few as 10 circulating B lymphocytes per microliter (0.4% of lymphocytes) can seroconvert (49, 50). Our regression analysis suggests the percentage of pre-vaccination naïve B lymphocytes as a better marker for successful vaccination than the total B lymphocytes since its association with seroconversion probability is independent not only of the percentages of total B lymphocytes but also of the rituximab exposure status, although its use falls short on the lack of standard normal value. Further analysis of other B lymphocytes subpopulations with a good correlation with vaccine responses (e.g., HLA-DR-hi, CXCR-hi, CD95-low, CD21-low B lymphocytes) (51) and its interactions with other rituximab-affected components of the immune system may be required to comprehensively analyze the key elements that drive the immune response to vaccination for IMDD patients. As predicting vaccination response *via* immunophenotyping is a work in progress, the use of rituximab-to-vaccination interval to determine an appropriate vaccination timeframe for rituximab-exposed IMDD patients is a more pragmatic solution for the status quo.

## 5 Conclusion

In conclusion, this study proposed a nine-month rituximab-to-vaccination interval as a time point that maximizes the immunological benefits of COVID-19 vaccines while avoiding unnecessary delay in vaccination and rituximab treatment for IMDD patients. A larger study may be warranted to confirm our finding as this is a relatively small single-centered study.

## Data availability statement

The raw data supporting the conclusions of this article will be made available by the authors, without undue reservation.

## Ethics statement

The study involving de-identified data of human participants was reviewed and approved by the Human Research Ethics Committee, Faculty of Medicine Ramathibodi Hospital, Mahidol University, which granted a waiver of consent. 

## Author contributions

All authors conceptualized and planed the study methodology. CS-a, YR, SC, TS, CS and PO-C collected the data. CS-a and YR performed data curation and conducted formal analysis. CS-a created data visualisation and wrote the original draft of the manuscript. KC, PR, PS, PO-C, and PP supervised the project administration. All authors contributed to the article and approved the submitted version.
